# The Effect of 2016 Chinese second-child policy and different maternal age on pregnancy outcomes in Hebei Province, China

**DOI:** 10.1186/s12884-023-05552-2

**Published:** 2023-04-19

**Authors:** Mei-Ling Tian, Guo-Juan Ma, Li-Yan Du, Ying Jin, Cui Zhang, Yuan-Ge Xiao, Zeng-jun Tang

**Affiliations:** 1 Department of Obstetrics and Gynecology, Hebei General Hospital, Shijiazhuang, China; 2 Department of Information Management, Hebei Center for Women and Children’s Health, Shijiazhuang, China

**Keywords:** Adverse pregnancy outcomes, Hebei, Age, Maternal, Complications

## Abstract

**Objective:**

To explore the effect of the 2016 Chinese second child policy and different maternal ages on adverse perinatal outcomes.

**Methods:**

Clinical data were collected from 22 monitoring hospitals in Hebei Province from January 1, 2013, to December 31, 2021. A total of 413,892 parturient were divided into 3 groups based on delivery age: 20–34, 35–39, and 40–55 years old. The clinical data were analyzed to explore the relationship among the 2016 Chinese second-child policy, maternal age, and various pregnancy risks.

**Results:**

Pregnancy complications showed an upward trend from 2013 to 2021.The top 10 incidences of pregnancy complications in Hebei Province were anemia, small for gestational age (SGA), large for gestational age (LGA), macrosomia, gestational diabetes mellitus (GDM), premature delivery, preeclampsia (PE), postpartum hemorrhage (PPH), placenta previa, and placental abruption. The two-child policy was implemented in 2016. The incidence of pregnancy complications, anemia, GDM, PE, placental abruption, cesarean delivery, premature delivery, SGA, LGA, macrosomia in 2016–2021 was significantly higher than that in 2013–2015 (*P*<0.05), and the proportion of women of advanced maternal age (AMA, ≥ 35 years old) increased from 2013 to 2021. Advanced maternal age was a risk factor for most assessed adverse pregnancy outcomes, including GDM, PE, placenta previa, placenta abruption, cesarean delivery, PPH, premature delivery, SGA, LGA and macrosomia.

**Conclusion:**

After the adjustment of the “second-child” policy, the incidence of pregnancy complications increased. Moreover, the risk of adverse pregnancy outcomes in AMA has increased. Early prevention and intervention should be implemented to cope with the occurrence of adverse perinatal outcomes.

## Introduction

Pregnancy is a special physiological period for women, during which a series of changes take place in the endocrine system, circulatory system, and others. During pregnancy, women are prone to complications. With the implementation of China’s “second-child” policy in 2016 and the change in attitudes toward AMA, the ages of pregnant women are trending upward [1, 2]. Advanced maternal age has been defined to describe women who are 35 years or older on the estimated date of delivery [2–5]. The proportion of AMA in China has risen from 2.96% to 1996 to 8.56% in 2007, and 10.1% in 2011 [6, 7], and the proportion of AMA in Beijing has risen to 14% as of 2016 [8]. Women with AMA may have an increased risk for maternal and infant complications during pregnancy including: preeclampsia (PE), gestational diabetes mellitus (GDM), postpartum hemorrhage (PPH), fetal growth restriction (FGR), placental abruption, and preterm birth [3, 9–11]. In addition, a systematic review and meta-analysis showed that AMA was associated with an increased risk of cesarean section [4, 12], and stillbirth [5, 13]. In this context, we aimed to evaluate the effect of the 2016 Chinese second child policy and different maternal ages on adverse perinatal outcomes in Hebei Province China from 2013 to 2021.

## Materials and methods

### Study area

Hebei Province is located between 113 ° 27? and 119 ° 50? E and 36 ° 05? and 42 ° 40? N. The region is an important grain and cotton producing area in China.

### Data collection

**Fig. 1:**
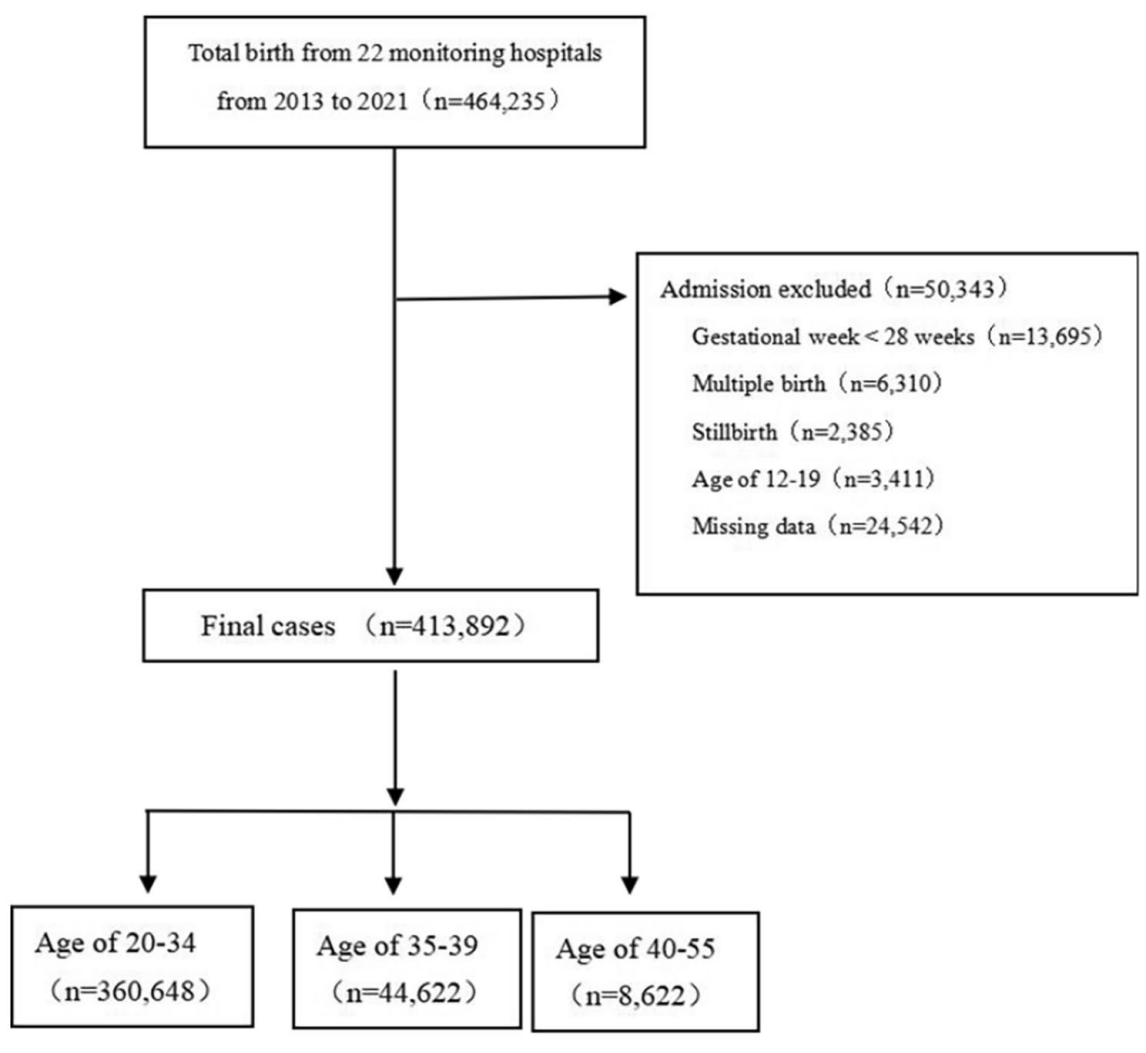
The flow chart of cases enrollment

This is a retrospective study. 413,892 delivery data from the monitoring information management system for pregnant women in 22 hospitals of Hebei Province China were collected from January 1, 2013 to December 31, 2021. We informed consent from all subjects. All methods were performed in accordance with the relevant regulations. Inclusion criteria are single live birth and over 28 weeks of gestation. Exclusion criteria included age<20y, stillbirth, multiple births, and incomplete data. The screening flow chart was shown in Fig. 1.

### Definition of variables

Based on the delivery age of the pregnant woman, the parturient were divided into 3 groups: 20–34, 35–39, and 40–55 years old. GDM was diagnosed if one or more thresholds were met or exceeded: Fasting blood glucose: 5.1 mmol/L, blood glucose at 1 h: 10.0 mmol/L, and blood glucose at 2 h: 8.5 mmol /L in the 75 g oral glucose tolerance test at 24–28 weeks.PE was defined as gestational hypertension with proteinuria of ≥ 300 mg in 24 h, or if there was no 24-hour collection, dipstick analysis of midstream or catheter urine specimens showed two readings of at least ++. PPH refers to the amount of bleeding exceeding 500 ml (after spontaneous delivery) or 1000 ml (after cesarean section) within 24 h after delivery. Premature delivery refers to the labor before 37 weeks. And SGA was defined as birth weight lower than the 10th percentile or two standard deviations below the average weight of babies of the same gestational age. LGA refers to birth weight greater than 90% of the average weight of the same gestational age. Macrosomia refers to the birth weight greater than 4000 g.

### Statistical analyses

SPSS 21.0 software was used for statistical analyses. The data description was presented as mean ± standard deviation (Mean ± SD) for continuous variables. The counting data are expressed in percentage (%). χ2-test is used for the comparison between groups of counting data. After adjusting the confounding factors, Multivariable logistic regression analysis was performed for the prediction of each pregnancy outcome. the criterion for statistical significance is α = 0.05.

## Results

**Fig. 2:**
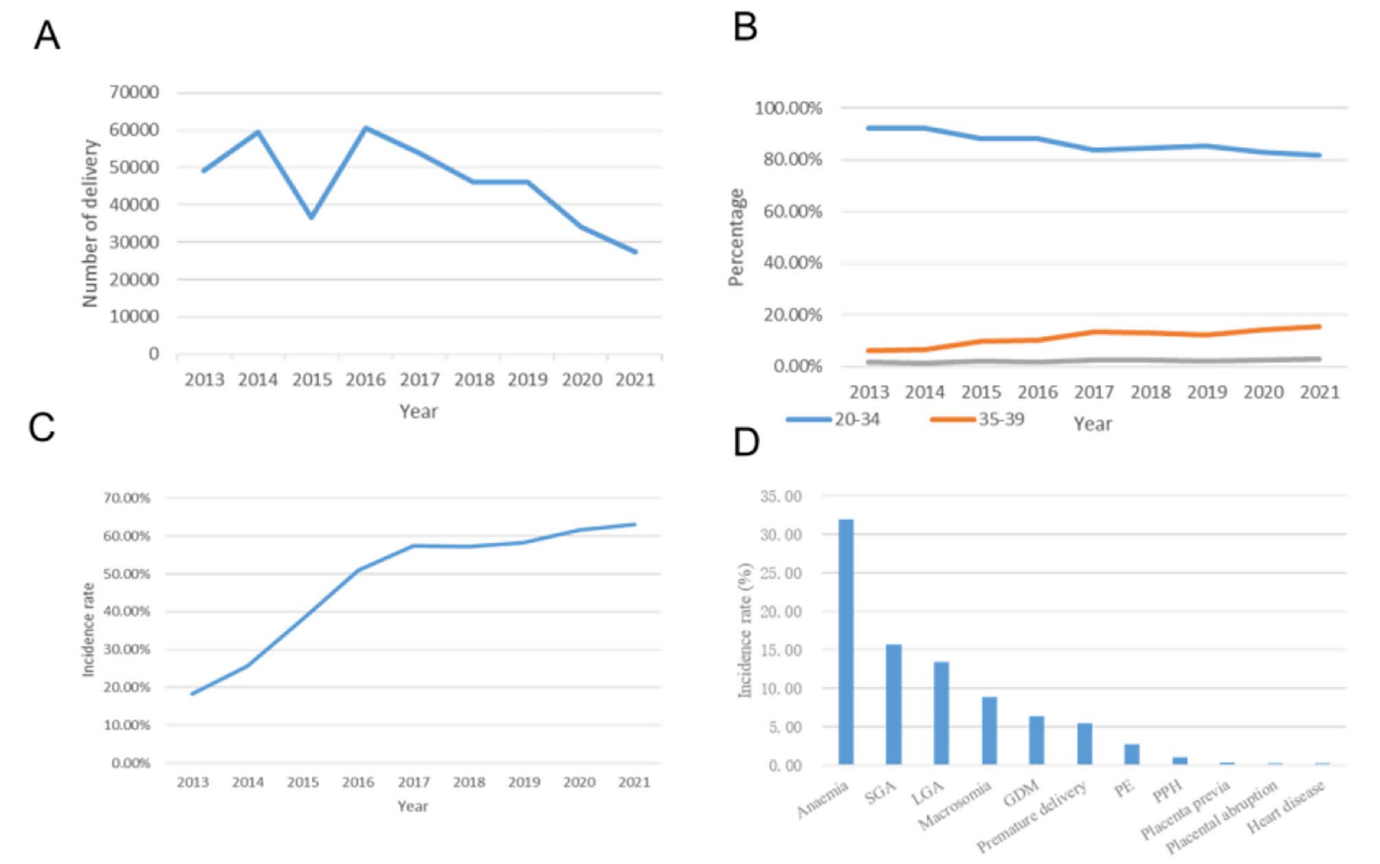
**(A)** Number of deliveries per year, **(B)** Percentage by age **(C)** Incidence rates of complications during pregnancy per year, **(D)** The ranking of different complication rates

During the study period, 413,892 deliveries were attended in our study. The total number of deliveries per year varied. In 2015, the number decreased sharply, and in 2016, it increased significantly; after 2016, it showed a downward trend (Fig. 2A). The two-child policy was implemented in 2016. The incidence of pregnancy complications, anemia, GDM, PE placental abruption, cesarean delivery, premature delivery, SGA, LGA, and macrosomia from 2016 to 2021 was significantly higher than that in 2013–2015 (*P*<0.05) (Table 1).

**Table 1:** The comparison of the maternal and neonatal outcomes between the period before and after the implementation of ‘second child policy’

	2013–2015 year	2016–2021 year	χ2	*P* Value
**Maternal**				
pregnancy complication	38,390(26.4%)	153,586(57.2%)	36056.019	<0.001
Anaemia	22,219(15.3%)	110,044(41.0%)	28710.2	<0.001
GDM	3825(2.6%)	23,028(8.6%)	5504.834	<0.001
PE	3652(2.5%)	7441(2.8%)	24.782	<0.001
Placenta previa	572(0.4%)	1143(0.4%)	2.434	0.119
Placental abruption	339(0.2%)	713(0.3%)	3.955	0.047
Heart disease	347(0.2%)	717(0.3%)	3.007	0.083
Cesarean delivery	76,160(52.4%)	141,054(52.6%)	1.47	0.225
PPH	832(0.6%)	3504(1.3%)	489.65	<0.001
**Infant**				
Premature delivery	7377(5.1%)	15,362(5.7%)	77.28	<0.001
Birth weight of newborn			32.449	<0.001
SGA	22,170(15.2%)	42,354(15.8%)		
LGA	19,376(13.3%)	36,522(13.6%)		
Macrosomia	13,272(9.1%)	23,733(8.8%)	9.181	0.002

The average delivery age was 29.21 ± 4.50 years. The proportion of different age groups changed, and the proportion of AMA increased (Fig. 2B). The incidence of complications in pregnant women in Hebei Province also showed an upward trend from 2013 to 2021 (Fig. 2C). The top 10 incidences of pregnancy complications in Hebei Province from 2013 to 2021 were anemia, SGA, LGA, macrosomia, GDM, premature delivery, PE, PPH, placenta previa, and placental abruption (Fig. 2D).

### Frequency of pregnancy complications according to maternal age group

**Fig. 3:**
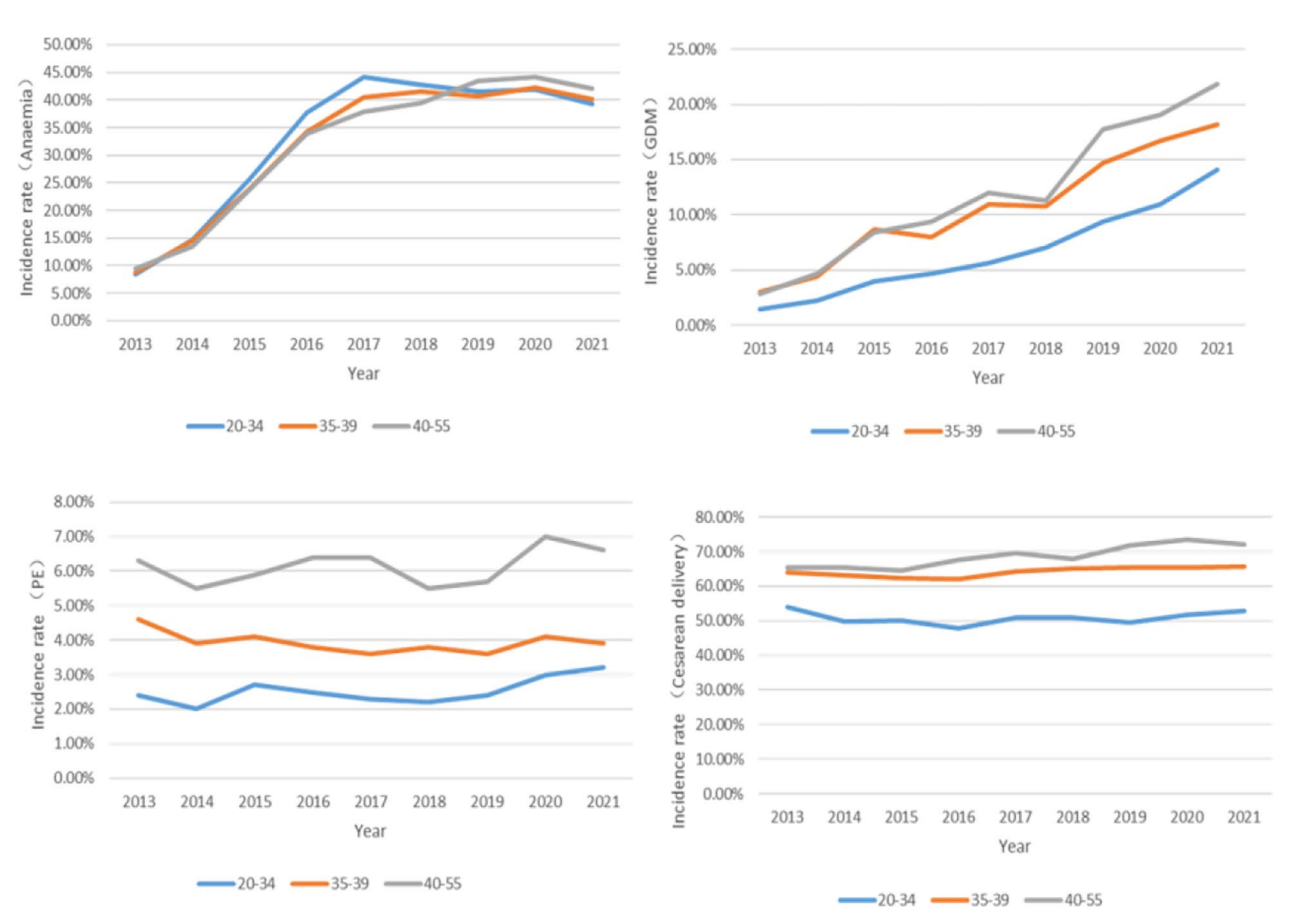
Maternal Outcomes in pregnant women of different ages from 2013 to 2021

**Fig. 4:**
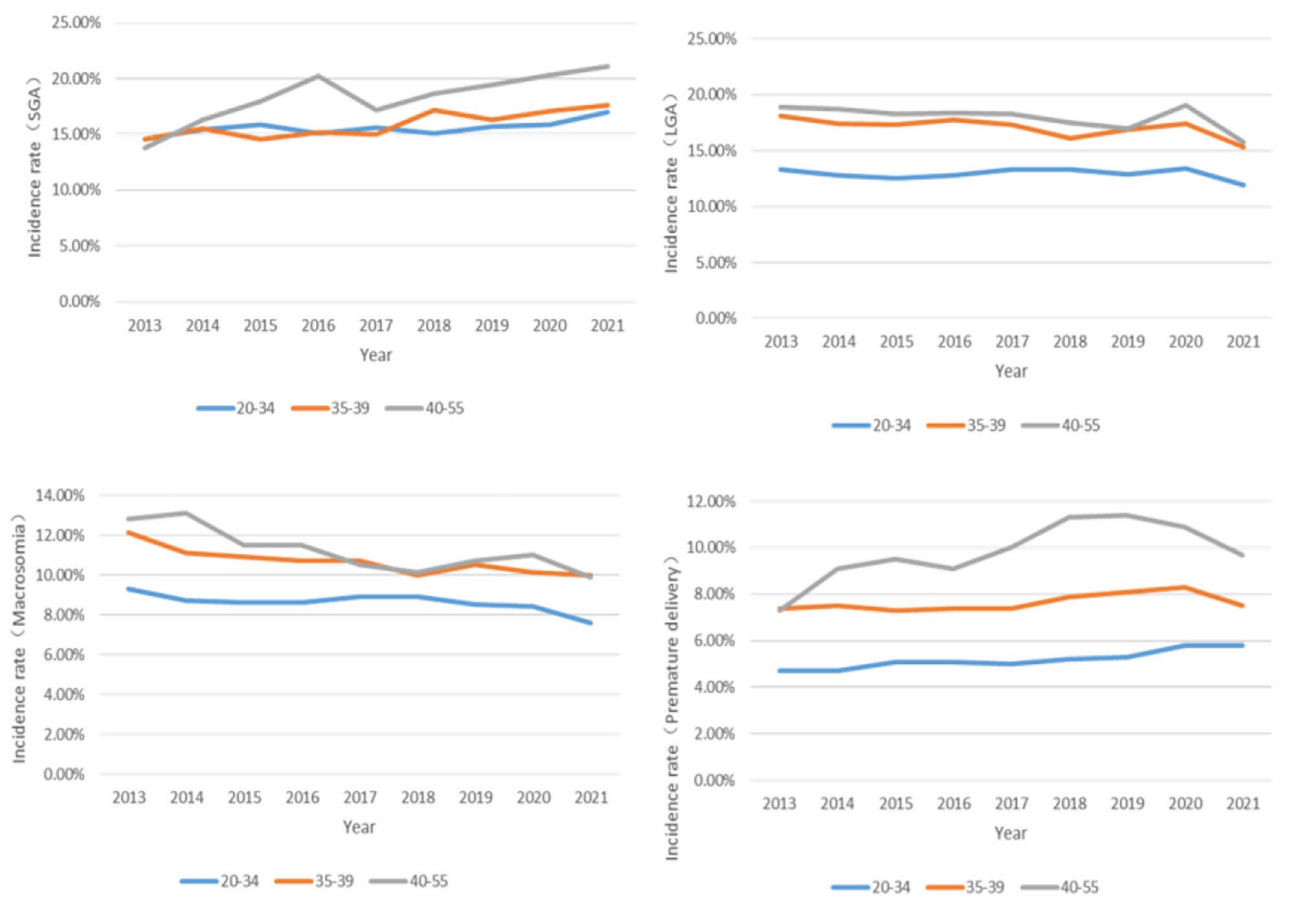
Infant Outcomes in pregnant women of different ages from 2013 to 2021

From 2013 to 2021, the frequency of GDM, PE, cesarean delivery, SGA, LGA, macrosomia, and premature delivery in the AMA group was always higher than that in the 20–34 age group per year. The incidence rate of anemia in the 20–34 age group was higher than that in the AMA group from 2013 to 2018, and lower from 2019 to 2021 (Figs. 3 and 4).

The total incidence of pregnancy complications in AMA individuals increased significantly. AMA individuals were at significantly increased risk of adverse pregnancy outcomes, including anemia, GDM, PE placenta previa, placental abruption, and cesarean delivery (*P*<0.05). There was no significant difference in heart disease, or PPH (*P*>0.05) (Table 2). For infant outcomes, there were significant differences in premature delivery, SGA, LGA and macrosomia (*P*<0.05) (Table 2) .

**Table 2:** Adverse Outcomes of Individuals of different ages(year) in Hebei, from 2013 to 2021[N (%)]

	20–34	35–39	40–55	χ2	*P* Value
**Maternal**					
Pregnancy complications	163,131(45.2%)	23,974(53.7%)	4871(56.5%)	1514.065	<0.001
Anaemia	114,105(31.60%)	15,290(34.30%)	2868(33.30%)	132.928	<0.001
GDM	20,878(5.80%)	4932(11.10%)	1043(12.10%)	2270.115	<0.001
PE	8835(2.40%)	1729(3.90%)	529(6.10%)	712.195	<0.001
Placenta previa	1268(0.40%)	364(0.80%)	83(1.00%)	271.468	<0.001
Placental abruption	869(0.20%)	148(0.30%)	35(0.40%)	20.890	<0.001
Heart disease	926(0.30%)	114(0.30%)	24(0.30%)	0.158	0.924
Cesarean delivery	182,608(50.60%)	28,669(64.20%)	5937(68.90%)	3898.796	<0.001
PPH	3771(1.00%)	463(1.00%)	102(1.20%)	1.582	0.453
**Infant**					
Premature delivery	18,467(5.10%)	3417(7.70%)	855(9.90%)	824.013	<0.001
Birth weight of newborn				849.882	<0.001
SGA	55,826(15.50%)	7114(15.90%)	1584(18.4%)		
LGA	46,745(13.00%)	7600(17.00%)	1553(18.00%)		
Macrosomia	31,328(8.70%)	4720(10.60%)	957(11.10%)	224.844	<0.001

**Fig. 5:**
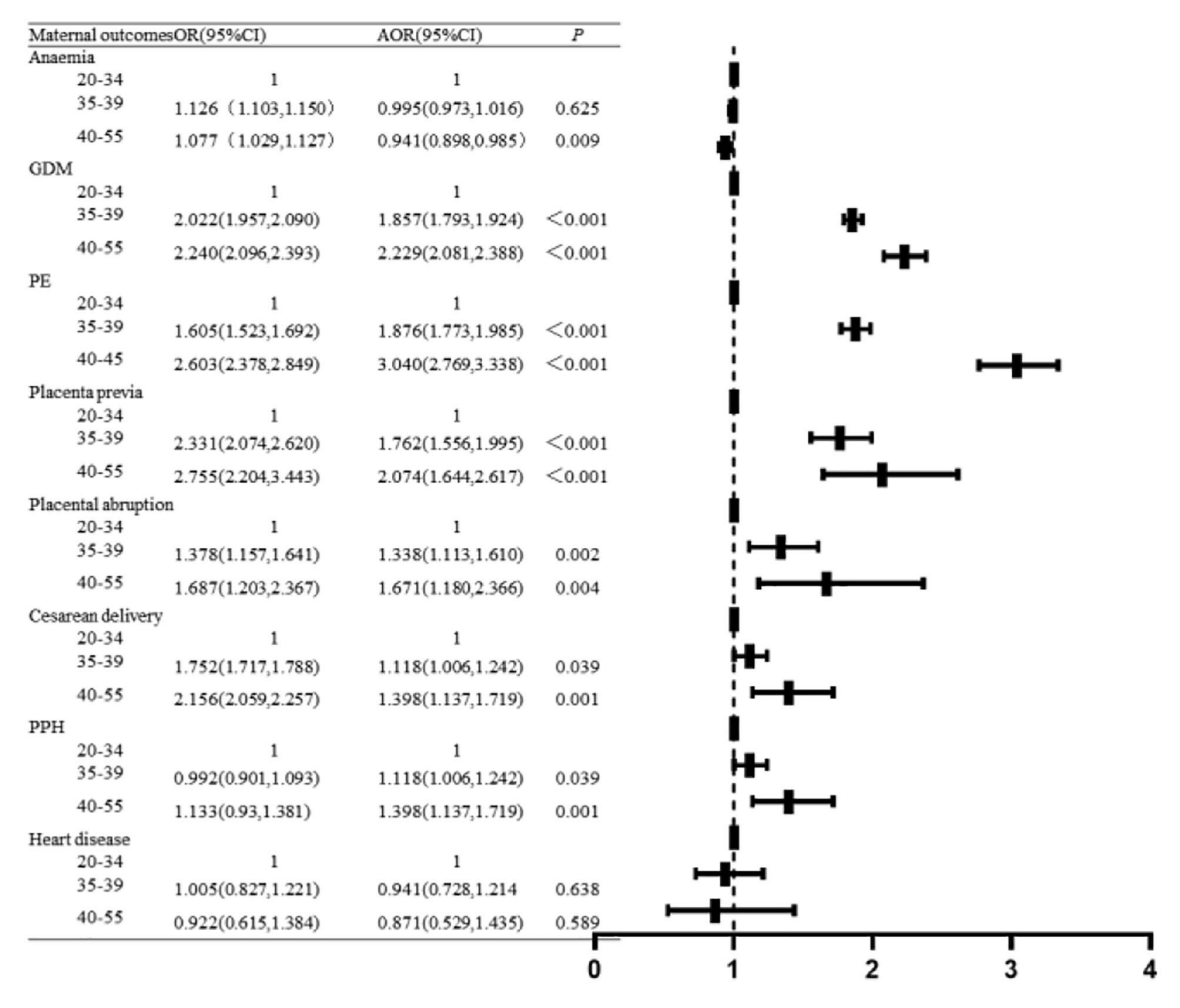
Univariate and multivariate logistic regression analysis model of maternal outcomes of individuals at different ages

**Fig. 6:**
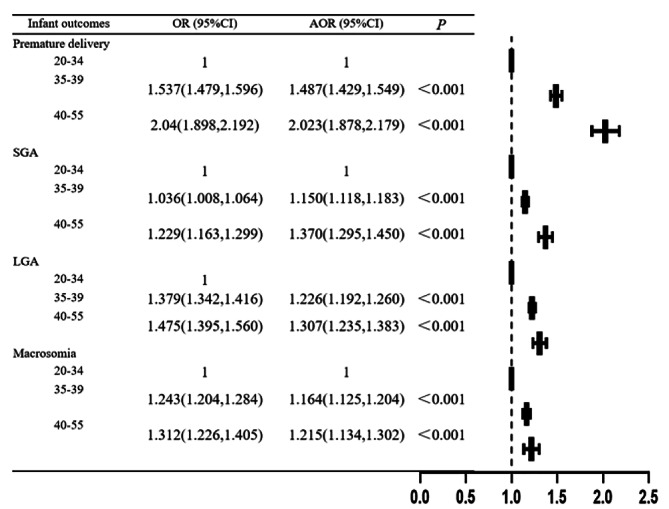
Univariate and multivariate logistic regression analysis model of infant outcomes of individuals at different ages

Individuals aged 40–55 and 35–39 were at significantly increased risk of most assessed adverse pregnancy outcomes, including GDM, PE, placenta previa, placenta abruption, cesarean delivery, PPH, premature delivery, SGA, LGA and macrosomia. Conversely, individuals aged 40–55 had a significantly lower risk of anemia. The analyses showed no difference in anemia for the 35–39 age group or heart disease for the both 35–39 and 40–55 age groups compared with 20–34 age group (Figs. 5 and 6).

## Discussion

In this study, the number of births decreased significantly in 2015, which may be related to 2015 being the Year of the Goat in China. The decision to not have children may follow traditional culture; many Chinese people prefer not to have children in the Year of the Goat, but instead prefer to have a “dragon baby” or a “monkey baby”. In addition, in 2016, the number of deliveries increased and the proportion of AMA women increased. This may be closely related to the recovery of the suppressed childbearing demand in the Year of the Goat and the implementation of the “second-child” policy. Moreover, with the rapid economic development in recent years, women of childbearing age have more freedom to postpone pregnancy. In addition, the use of assisted reproductive technology increases the chance of pregnancy for patients with primary and secondary infertility, but also increases the average age of pregnant women.

Pregnancy complications showed an upward trend from 2013 to 2021. After the implementation of the “second-child policy”, the incidence of pregnancy complications also increased. The top 10 incidences of pregnancy complications in Hebei Province from 2013 to 2021 are anemia, SGA, LGA, macrosomia, GDM, premature delivery, PE, PPH, and placenta previa. The incidence of pregnancy complications (such as anemia, PE, GDM, and SGA) increased with age, which is consistent with previous studies [6, 9, 14–17]. The age range of 40–55 years is a protective factor for anemia, which may be related to their attention to nutrition and iron supplement during pregnancy. Importantly, the incidence of macrosomia decreased, which may be related to the importance of screening and early management of GDM to avoid the occurrence of macrosomia. A rise of maternal blood glucose causes hyperinsulinemia in the fetus, Insulin cannot be transported through the placenta, which promotes the deposition of liver glycogen, protein synthesis, and fat deposition in the fetus, thereby promoting growth and development [12, 18]. Early screening and management of GDM can help patients obtain stable blood sugar, which may decrease the development of macrosomia and neonates [7, 19].

In this study, we demonstrate that AMA is associated with the occurrence of GDM. With increasing age, the metabolic capacity of women’s bodies decreases, and the growth of fat is more significant. As body mass index gradually increases, the risk of hypertensive disorder and diabetes, which complicate pregnancy, also increase [8, 20]. The risk of PE, GDM, and PPH in obese women is higher than that in women with normal body mass [8, 20]. Moreover, an increase in age can lead to insulin resistance, insulin secretion and insulin receptor abnormalities. The probability of abnormal blood glucose diseases in AMA pregnant women is therefore higher than in younger women. A growing number of studies have suggested that hyperglycemia increases the risk of PE [9, 21, 22]. Abnormal lipid metabolism in patients with hypertension is easily complicated by abnormal glucose metabolism. Similarly, AMA is a risk factor for PE. Therefore, for AMA women, there should be an increased focus on nutrition management during pregnancy to avoid metabolic diseases.

In addition, we also found AMA increased the risk of cesarean delivery, and PPH. With increasing age, physical strength decreases in women. Uterine atony may occur during delivery, and the risk of postpartum hemorrhage and conversion to cesarean section due to a prolonged labor process also increases [23]. Therefore, we should consider energy supplementation in the labor process of older women to avoid uterine atony and reduce the risk of postpartum hemorrhage and cesarean section. Moreover, we found that AMA was a risk factor for placental abruption and placenta previa. Consistent with previous studies, an increase in age significantly increased the risk of both outcomes [11, 24–26].

Also consistent with previous studies [3, 9, 12], we found that women of AMA were at higher risk of premature birth before 37 weeks of gestation. The incidence of SGA babies was significantly higher, which may be related to the increase of PE in AMA pregnant women. Moreover, we also found the incidence of macrosomia and LGA in the offspring of women of AMA was significantly higher than that of the offspring of younger mothers. In recent years, the incidence of GDM in pregnant women has gradually increased. The risk of GDM in AMA women increased. Previous studies [27–29] showed that the risk of GDM pregnant women delivering macrosomia and LGA infants was significantly higher than that of non-GDM pregnant women. This could also be related to the fact that AMA mothers pay increased attention to nutritional supplementation during pregnancy, resulting in excessive nutrition.

After the introduction of the “three-child” policy, the delivery volume will likely continue to increase. The increasing proportion of women of AMA should attract the attention of obstetricians, and screening for basic diseases before pregnancy should be performed. Nutritional guidance should be provided in the first trimester of pregnancy and vitamins should be administered reasonably to avoid complications. The second and third trimesters of pregnancy should screen for and treat pregnancy complications to reduce the occurrence of adverse pregnancy outcomes and increase safety for mothers and infants as much as possible.

However, our study had some limitations. First, this is a retrospective study. Second, Due to the limitation of the reported data, the reason for cesarean section is not included in the data, so we can’t screen out the cases of elective CS. Therefore, our cases do not exclude cases of elective CS. This is the limitation of our article data. Third, this study lacks clinical data of pregnant women’s height, weight and weight gain during pregnancy. and a review of other factors including diet, lifestyle factors and family personal history. These limitations may lead to analyzed biases. Future studies will include a more detailed investigation by involving a more comprehensive list of factors.

## Data Availability

The datasets used in the current study are available from the corresponding author on reasonable request.

## Abbreviations

SGA: Small for Gestational Age
LGA: Large for Gestational Age
GDM: Gestational Diabetes Mellitus
PE: Preeclampsia
PPH: Postpartum Hemorrhage
AMA: Advanced Maternal Age
